# Supplementary figures and images for: CAMAP: Artificial neural networks unveil the role of codon arrangement in modulating MHC-I peptides presentation
Source: PLoS Comput Biol. 2021 Oct 22;17(10):e1009482. doi: 10.1371/journal.pcbi.1009482 (PMC8577786; doi:10.1371/journal.pcbi.1009482)

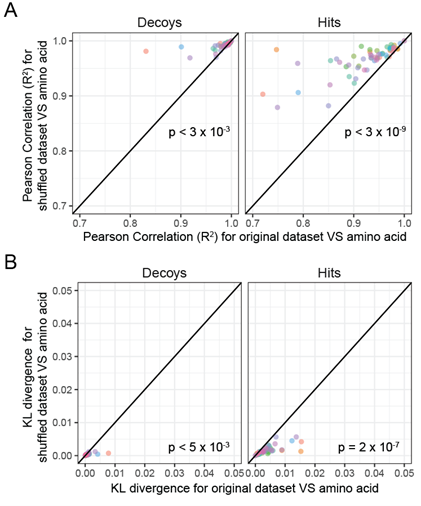

Supplement: S1 Fig — (A) Pearson correlation (R2) factors and (b) Kullback-Leibler (KL) divergence between positional distribution of codons and their corresponding amino acid in the shuffled (y axis) VS original (x axis) datasets. For all codons, the shuffled dataset showed greater correlations (A) and smaller KL divergence to their respective amino acid distributions than the original datasets (p < 1 x 10^−8^, assessed using unilateral paired Student T test). (TIF) [file pcbi.1009482.s001.tif]

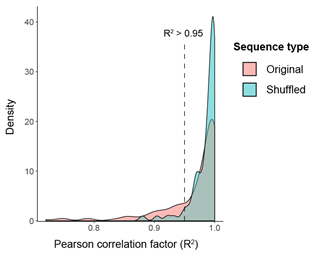

Supplement: S2 Fig — 92% of codons in the shuffled dataset reflecting the amino acids distribution with a R2 > 0.95, compared to only 69% in the original dataset (p < 5x10^-5^). (TIF) [file pcbi.1009482.s002.tif]

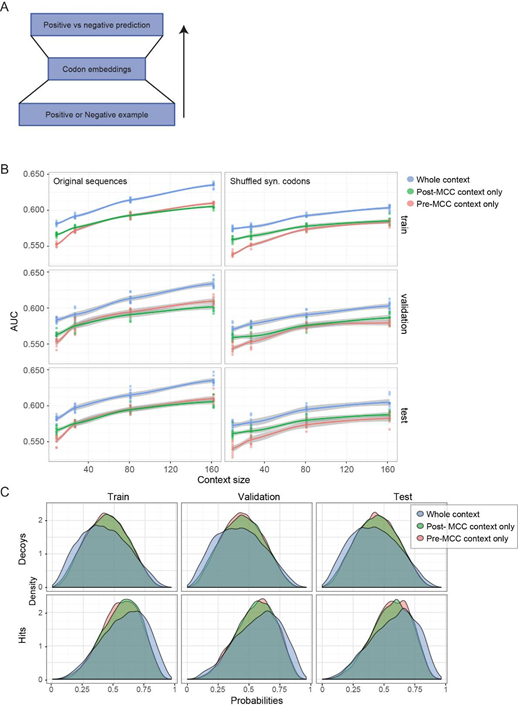

Supplement: S21 Fig — (A) Architecture of the ANN used in this work. (B) Results for the AUC on all train, validation and test subsets. Grey areas represent the 95% confidence intervals. (C) Distributions of output probabilities of CAMAPs used to calculate correlations in S22 Fig. (TIF) [file pcbi.1009482.s021.tif]

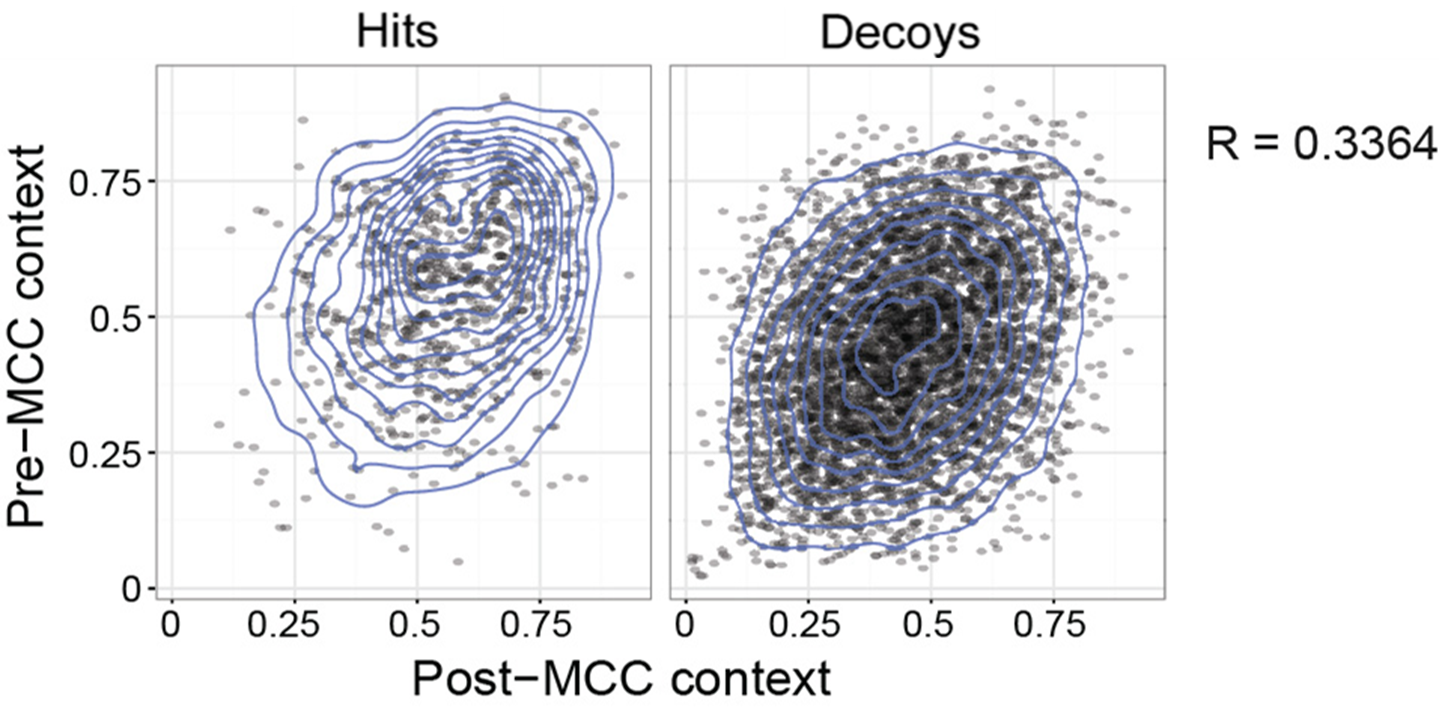

Supplement: S22 Fig — For each sequence in the test set we calculated the average prediction score given by CAMAPs in each condition, and calculated the Pearson correlation using the R software. Densities were calculated on all points and drawn using ggplot2. Only a random subset of the points is represented in the figures to limit their size. (TIF) [file pcbi.1009482.s022.tif]

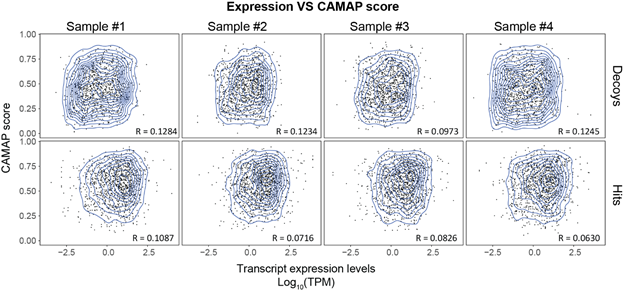

Supplement: S23 Fig — (TIF) [file pcbi.1009482.s023.tif]

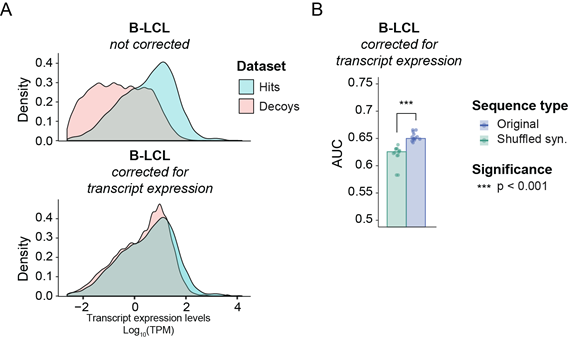

Supplement: S24 Fig — (A) Distribution of transcript expression levels for normal datasets (related to Fig 2) and the dataset used here to retrain CAMAP. As shown in this figure, the decoy dataset was selected to mirror the distribution of transcript expression level in the hit dataset. (B) CAMAP performance (measured by the AUC) when trained using the decoy dataset that mirrors the transcript expression levels of the hit dataset. Significance was assessed using bilateral paired Student T test (p = 5.36 x 10^−7^). (TIF) [file pcbi.1009482.s024.tif]

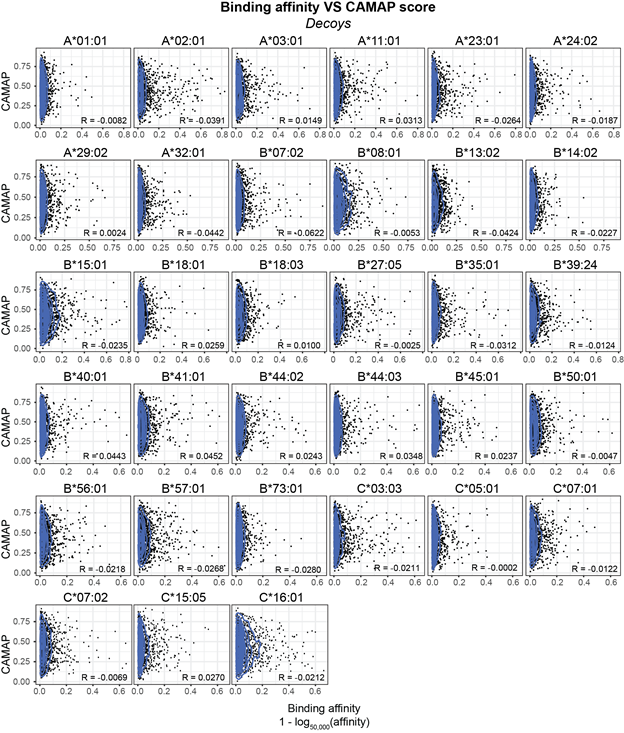

Supplement: S25 Fig — (TIF) [file pcbi.1009482.s025.tif]

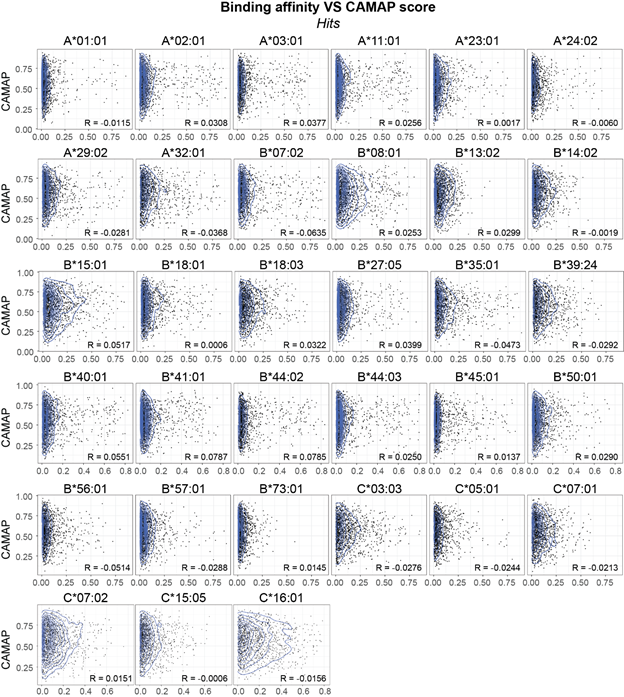

Supplement: S26 Fig — (TIF) [file pcbi.1009482.s026.tif]

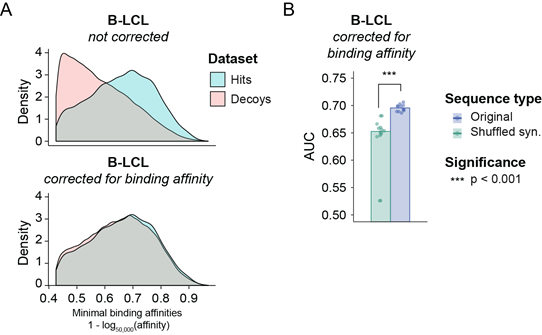

Supplement: S27 Fig — (A) Distribution of binding affinities for normal dataset (related to Fig 2) and the corrected dataset used to retrain CAMAP. As shown in this figure, the decoy dataset was selected to mirror the distribution of binding affinities in the hit dataset. (B) CAMAP performance (measured by the AUC) when trained using the decoy dataset that mirrors the binding affinities of the hit dataset. Significance was assessed using bilateral paired Student T test (p = 1.21 x 10^−9^). (TIF) [file pcbi.1009482.s027.tif]

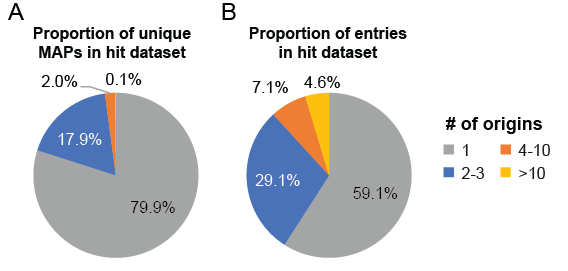

Supplement: S28 Fig — (A) Proportion of unique MAPs that can be ascribed to a single origin, 2–3, 4–10 or >10 possible origins. (B) Proportion of entries in the hit dataset that encode for MAPs with a single origin, 2–3, 4–10 or >10 possible origins (TIF) [file pcbi.1009482.s028.tif]

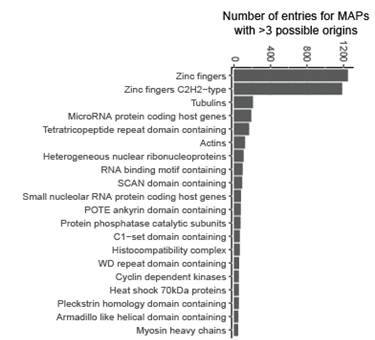

Supplement: S29 Fig — (TIF) [file pcbi.1009482.s029.tif]

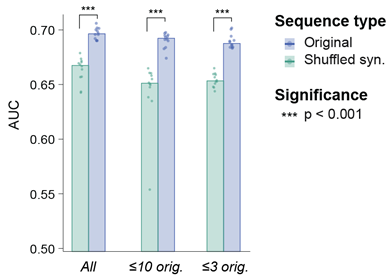

Supplement: S30 Fig — (TIF) [file pcbi.1009482.s030.tif]

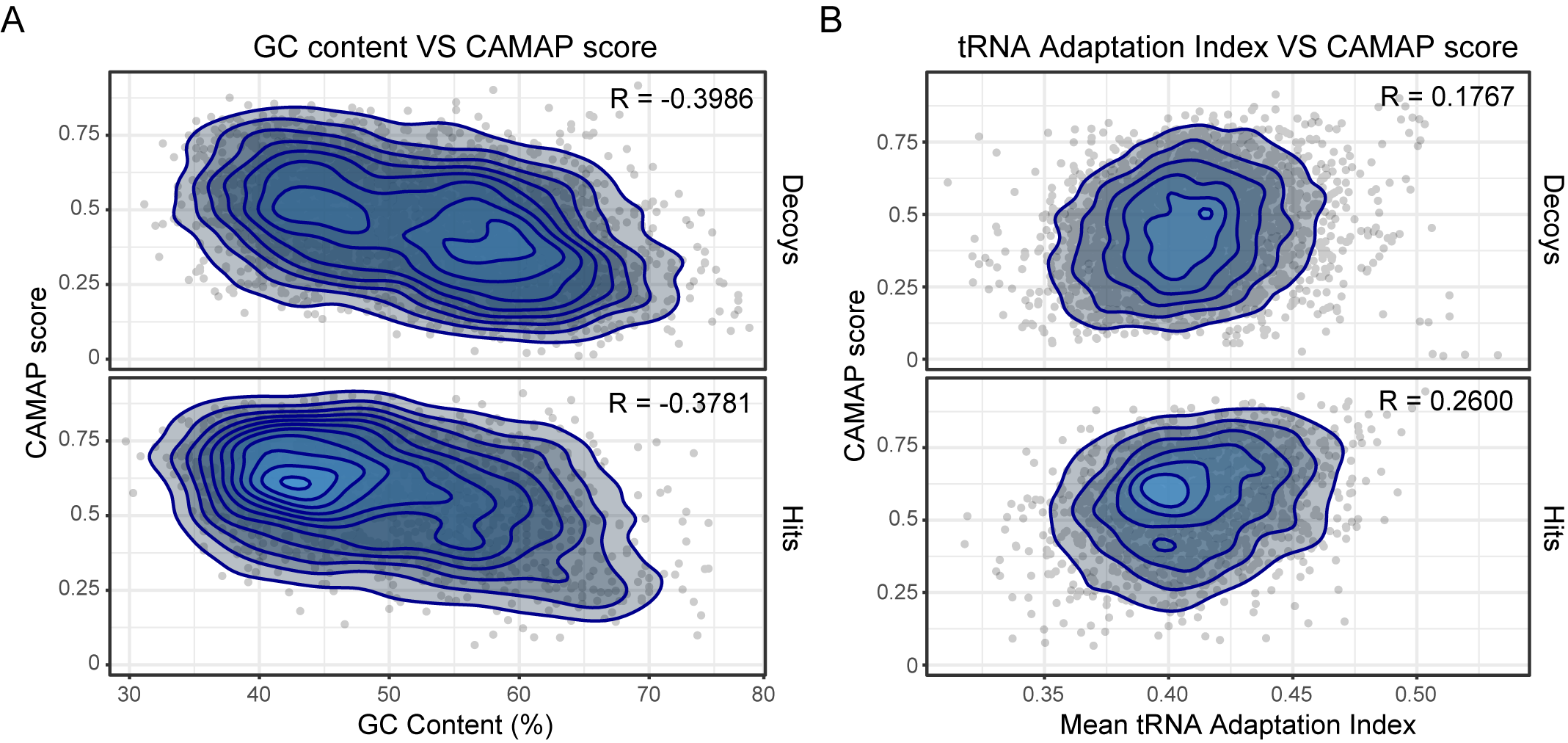

Supplement: S31 Fig — Pearson’s R correlation score is shown on the graphs. (TIF) [file pcbi.1009482.s031.tif]

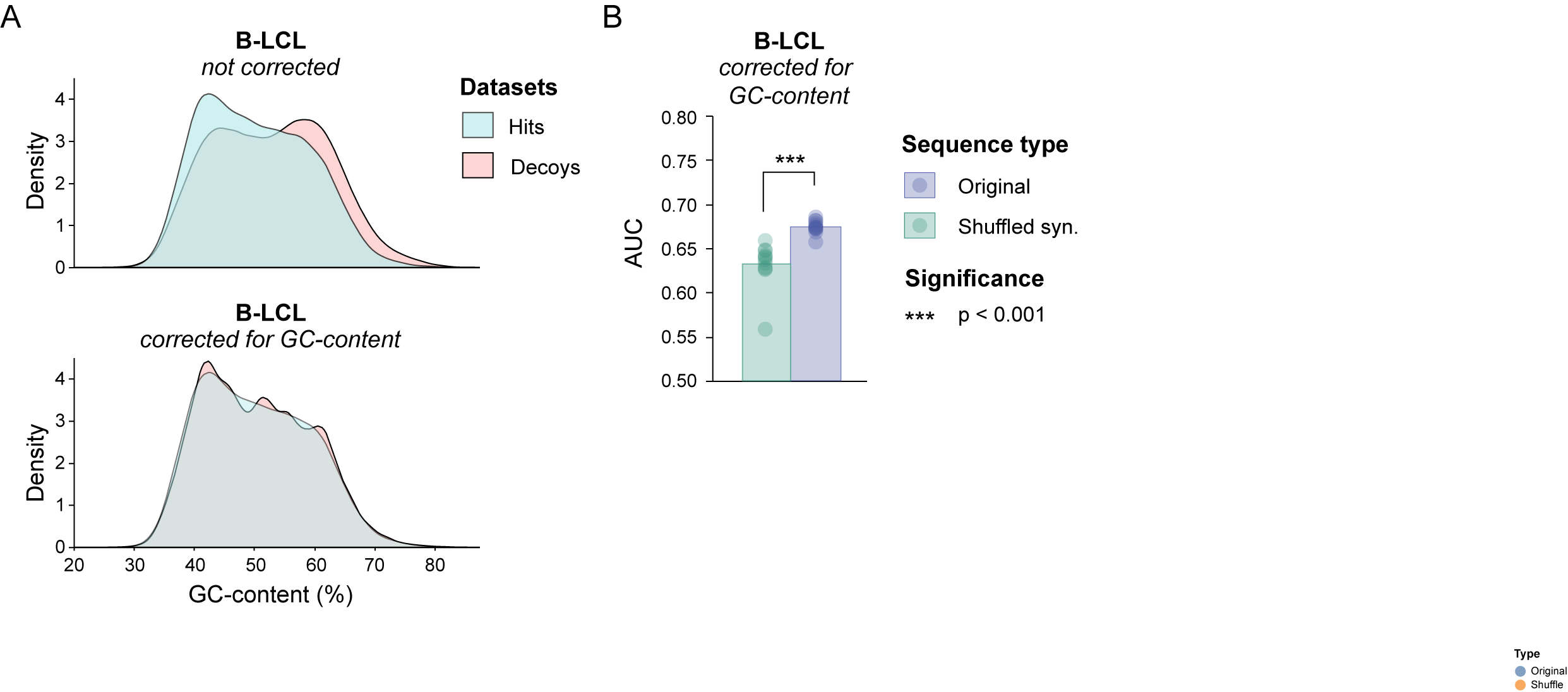

Supplement: S32 Fig — (A) Distribution of transcripts’ GC content for normal dataset (related to Fig 2) and the corrected dataset used to retrain CAMAP. As shown in this figure, the decoy dataset was selected to mirror the distribution of GC content in the hit dataset. (B) CAMAP performance (measured by the AUC) when trained using the decoy dataset that mirrors the GC content of the hit dataset. Significance was assessed using bilateral paired Student T test (p = 1.07 x 10^−4^). (TIF) [file pcbi.1009482.s032.tif]

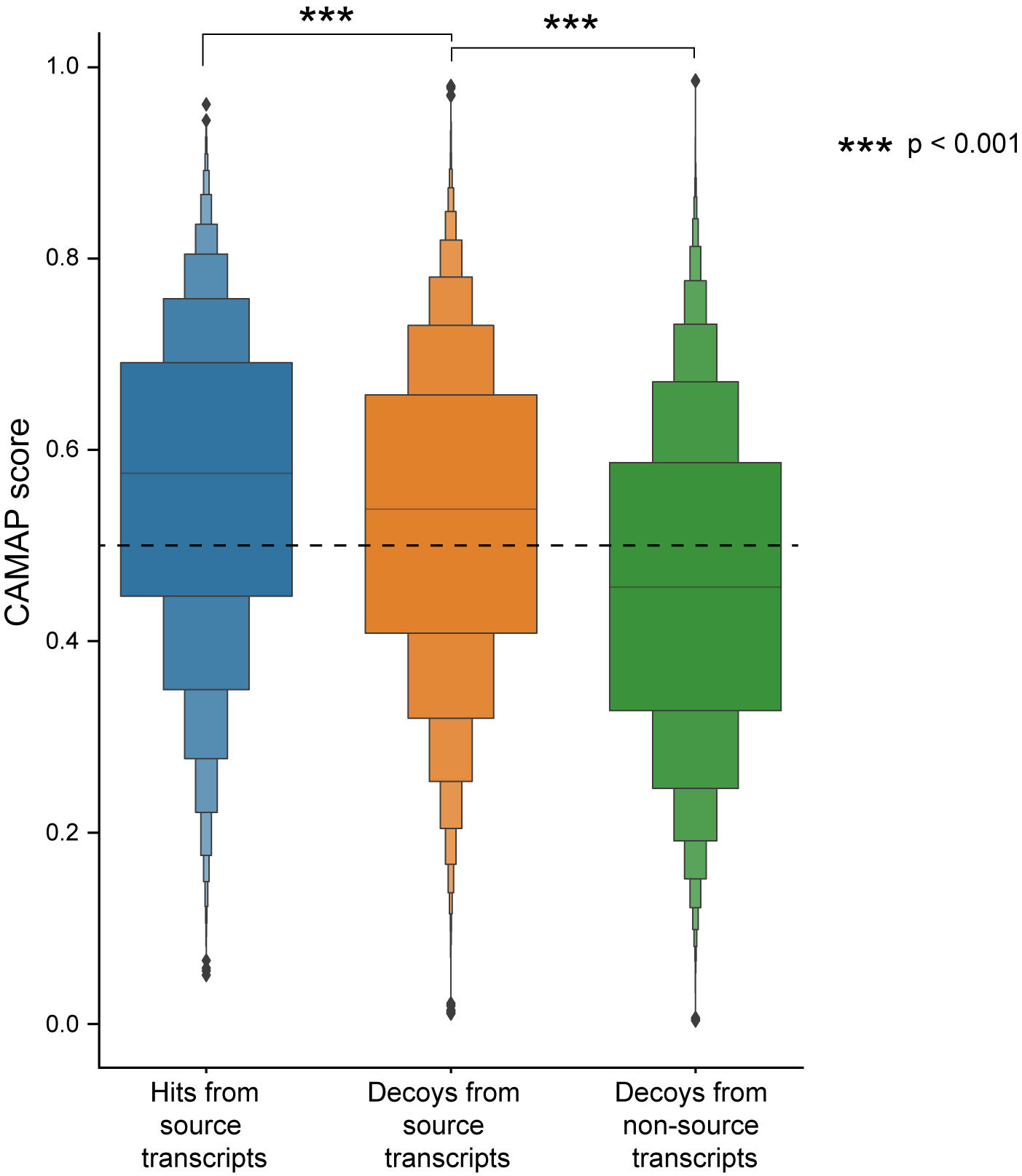

Supplement: S33 Fig — CAMAP was trained using hits from source transcripts and decoys from non-source transcripts only. Significance was assessed using bilateral unpaired T test. (TIF) [file pcbi.1009482.s033.tif]

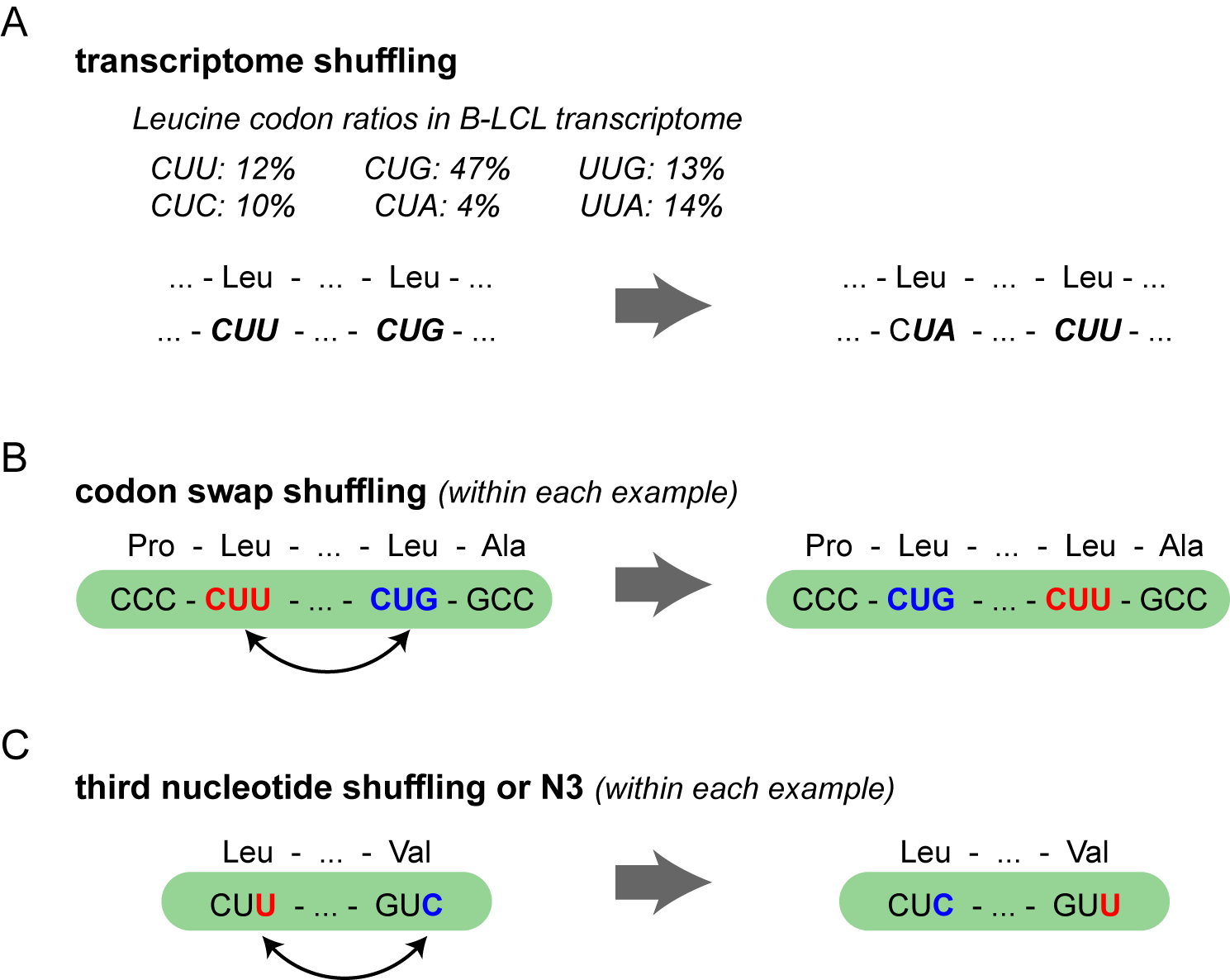

Supplement: S34 Fig — (A) Transcriptome shuffling: generates shuffled sequences by replacing each codon by one of its synonymous codons (including itself) according to the codon usage within the transcriptome, regardless of the codon used within each example. (B) Codon swap shuffling: synonymous codons present within a given example are swapped with one another. (C) Third nucleotide shuffling or N3: the third nucleotide of codons are swapped within each example to preserve amino acid sequences and GC content. Here, the global codon usage is completely different than normal codon usage in humans, as the frequency of codons is not taken into account during shuffling. (TIF) [file pcbi.1009482.s034.tif]

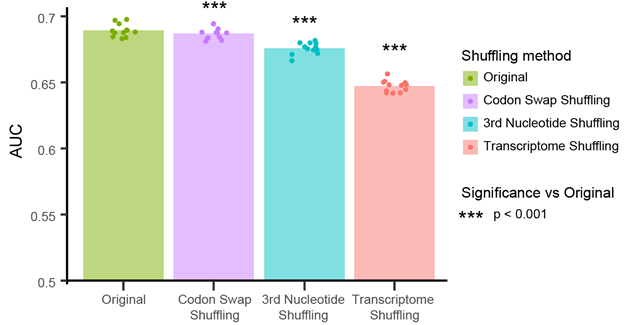

Supplement: S35 Fig — The performance of CAMAP networks (n = 12) pre-trained on original (non-shuffled) datasets was evaluated on three shuffled datasets according to the methods depicted in S34 Fig. Significance was assessed using a paired bilateral T test. (TIF) [file pcbi.1009482.s035.tif]

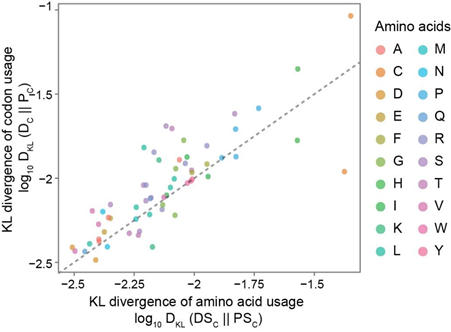

Supplement: S36 Fig — Shuffled sequences represent amino acid usage, as codon-specific information are removed with synonymous codon shuffling. (TIF) [file pcbi.1009482.s036.tif]

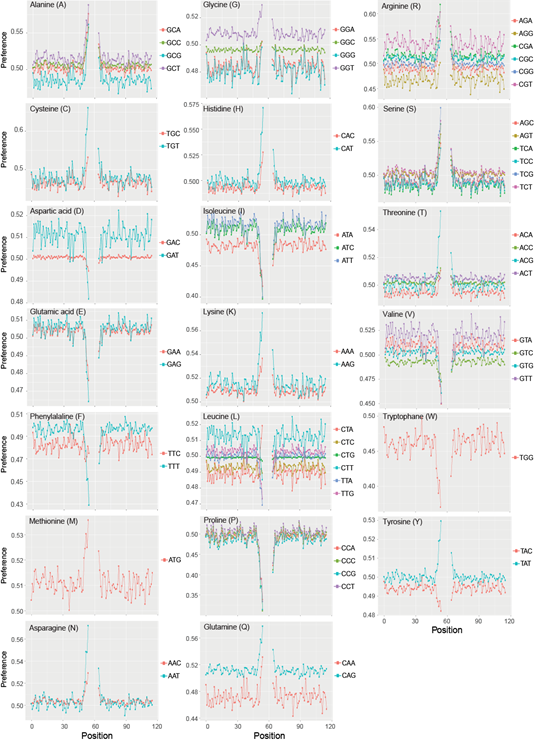

Supplement: S37 Fig — See Materials and Methods for more details. (TIF) [file pcbi.1009482.s037.tif]

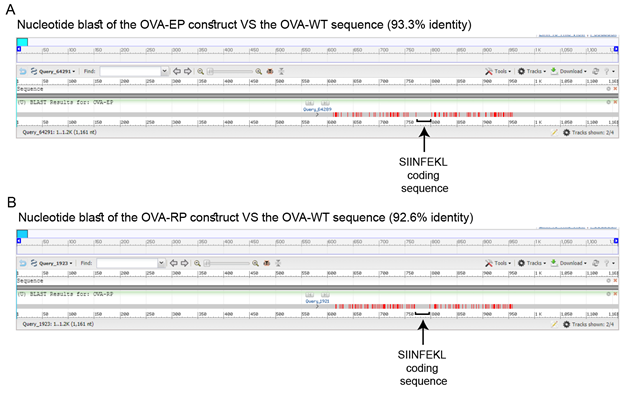

Supplement: S38 Fig — (A) Comparison of the OVA-EP nucleotide sequence to the wildtype OVA sequence. The OVA-EP and OVA-WT sequences have 93.3% nucleotide identity for a total of 78 modified nucleotides. (B) Comparison of the OVA-RP nucleotide sequence to the wildtype OVA sequence. The OVA-EP and OVA-WT sequences have 92.6% nucleotide identity, for a total of 86 modified nucleotides. Mutations, shown in red, are located only in the 162 nucleotide regions flanking the SIINFEKL coding codons. Of note, the SIINFEKL coding codons (nucleotides 772–799) were not modified between the 3 constructs. (TIF) [file pcbi.1009482.s038.tif]

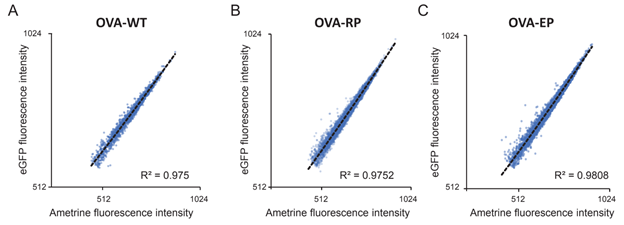

Supplement: S39 Fig — Single cell eGFP and Ametrine fluorescence intensities measured at 10 hours post-induction are shown for the OVA-WT (A), OVA-EP (B) and OVA-RP (C) constructs. N.B.: only transduced cells are shown (eGFP+ cells). (TIF) [file pcbi.1009482.s039.tif]

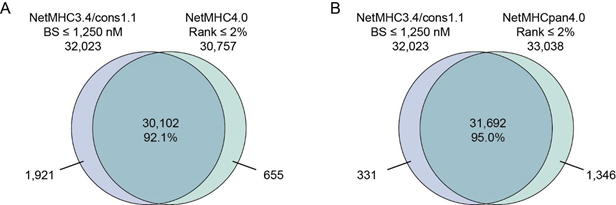

Supplement: S40 Fig — Validation of MHC-I associated peptides (MAP) dataset from Pearson H. et al. (2016) using the new versions of MAP binding affinity prediction algorithm NetMHC4.0 (A) and NetMHCpan4.0 (B). (TIF) [file pcbi.1009482.s040.tif]

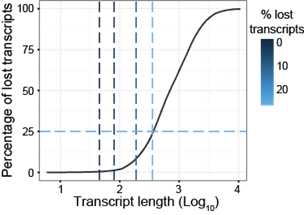

Supplement: S41 Fig — Transcript length corresponds to C x 2 + 27, where C is the context size in nucleotides and 27 the length of the MCCs. Related to Fig 1A. (TIF) [file pcbi.1009482.s041.tif]
