# Supplementary material for: CAMAP: Artificial neural networks unveil the role of codon arrangement in modulating MHC-I peptides presentation
Source: PLoS Comput Biol. 2021 Oct 22;17(10):e1009482. doi: 10.1371/journal.pcbi.1009482 (PMC8577786; doi:10.1371/journal.pcbi.1009482)
Supplement: S1 Table — SIINFEKL MCCs are shown in bold, while the variant regions (pre- and post-MCCs flanking sequences, context size of 162-nucleotides) are in blue and italics. Related to Fig 7. (DOCX) [file pcbi.1009482.s042.docx]

**Supplementary Table S1. Nucleotide sequences of the EP and RP constructs**. SIINFEKL MCC is shown in bold, while the variant regions (pre- and post-MCC contexts of 162-nucleotides) are in blue and italics. Related to Fig. 7.

| **OVA-EP** |
| --- |
| ATGGGCTCCATCGGTGCAGCAAGCATGGAATTTTGTTTTGATGTATTCAAGGAGCTCAAAGTCCACCATGCCAATGAGAACATCTTCTACTGCCCCATTGCCATCATGTCAGCTCTAGCCATGGTATACCTGGGTGCAAAAGACAGCACCAGGACACAAATAAATAAGGTTGTTCGCTTTGATAAACTTCCAGGATTCGGAGACAGTATTGAAGCTCAGTGTGGCACATCTGTAAACGTTCACTCTTCACTTAGAGACATCCTCAACCAAATCACCAAACCAAATGATGTTTATTCGTTCAGCCTTGCCAGTAGACTTTATGCTGAAGAGAGATACCCAATCCTGCCAGAATACTTGCAGTGTGTGAAGGAACTGTATAGAGGAGGCTTGGAACCTATCAACTTTCAAACAGCTGCAGATCAAGCCAGAGAGCTCATCAATTCCTGGGTAGAAAGTCAGACAAATGGAATTATCAGAAATGTCCTTCAGCCAAGCTCCGTGGATTCTCAAACTGCAATGGTTCTGGTTAATGCCATTGTCTTCAAAGGACTGTGGGAGAAAGCATTTAAGGATGAAGACACACAAGCAATGCCTTTCAGAGTGACTGAG*CAGGAGTCTAAGCCTGTTCAGATGATGTATCAGATTGGTCTTTTTCGTGTTGCTTCTATGGCTTCTGAGAAGATGAAGATTCTTGAGCTTCCTTTTGCTAGTGGTACTATGTCTATGCTTGTTCTTCTTCCTGATGAGGTTTCTGGTCTTGAGCAGCTTGAA***AGTATAATCAACTTTGAAAAACTG***ACTGAGTGGACTTCTTCTAACGTTATGGAGGAGCGTAAGATTAAGGTTTATCTTCCTCGTATGAAGATGGAGGAGAAGTATAACCTTACTTCTGTTCTTATGGCTATGGGAATTACTGATGTTTTTTCTAGTTCTGCTAACCTTAGTGGTATTTCTTCGGCT*GAGAGCCTGAAGATATCTCAAGCTGTCCATGCAGCACATGCAGAAATCAATGAAGCAGGCAGAGAGGTGGTAGGGTCAGCAGAGGCTGGAGTGGATGCTGCAAGCGTCTCTGAAGAATTTAGGGCTGACCATCCATTCCTCTTCTGTATCAAGCACATCGCAACCAACGCCGTTCTCTTCTTTGGCAGATGTGTTTCCCCTTAA |
| **OVA-RP** |
| ATGGGCTCCATCGGTGCAGCAAGCATGGAATTTTGTTTTGATGTATTCAAGGAGCTCAAAGTCCACCATGCCAATGAGAACATCTTCTACTGCCCCATTGCCATCATGTCAGCTCTAGCCATGGTATACCTGGGTGCAAAAGACAGCACCAGGACACAAATAAATAAGGTTGTTCGCTTTGATAAACTTCCAGGATTCGGAGACAGTATTGAAGCTCAGTGTGGCACATCTGTAAACGTTCACTCTTCACTTAGAGACATCCTCAACCAAATCACCAAACCAAATGATGTTTATTCGTTCAGCCTTGCCAGTAGACTTTATGCTGAAGAGAGATACCCAATCCTGCCAGAATACTTGCAGTGTGTGAAGGAACTGTATAGAGGAGGCTTGGAACCTATCAACTTTCAAACAGCTGCAGATCAAGCCAGAGAGCTCATCAATTCCTGGGTAGAAAGTCAGACAAATGGAATTATCAGAAATGTCCTTCAGCCAAGCTCCGTGGATTCTCAAACTGCAATGGTTCTGGTTAATGCCATTGTCTTCAAAGGACTGTGGGAGAAAGCATTTAAGGATGAAGACACACAAGCAATGCCTTTCAGAGTGACTGAG*CAAGAATCCAAACCGGTCCAAATGATGTACCAAATAGGGCTATTCAGGGTCGCGTCCATGGCGTCCGAAAAAATGAAAATACTAGAACTACCGTTCGCGTCAGGGACGATGTCCATGCTCGTCCTACTACCGGACGAAGTCTCCGGACTCGAACAACTCGAG***AGTATAATCAACTTTGAAAAACTG***ACAGAATGGACATCCTCCAATGTCATGGAAGAAAGGAAAATAAAAGTCTACCTCCCGAGGATGAAAATGGAAGAAAAATACAATCTAACATCCGTCCTAATGGCGATGGGTATAACAGACGTCTTCTCCTCATCCGCGAATCTATCAGGGATATCCAGCGCG*GAGAGCCTGAAGATATCTCAAGCTGTCCATGCAGCACATGCAGAAATCAATGAAGCAGGCAGAGAGGTGGTAGGGTCAGCAGAGGCTGGAGTGGATGCTGCAAGCGTCTCTGAAGAATTTAGGGCTGACCATCCATTCCTCTTCTGTATCAAGCACATCGCAACCAACGCCGTTCTCTTCTTTGGCAGATGTGTTTCCCCTTAA |
