# Supplementary material for: CAMAP: Artificial neural networks unveil the role of codon arrangement in modulating MHC-I peptides presentation
Source: PLoS Comput Biol. 2021 Oct 22;17(10):e1009482. doi: 10.1371/journal.pcbi.1009482 (PMC8577786; doi:10.1371/journal.pcbi.1009482)
Supplement: S2 Table — The lower the number of peptides needed to capture the respective number of epitopes, the better the performance of the prediction model. This is also illustrated by the percentage of false identification (false positive rate, FPR) reported here. Peptides were rank ordered according to regression scores. Of note, only the maximal transcript expression was used for peptides with multiple potential origins. (DOCX) [file pcbi.1009482.s043.docx]

**Supplementary Table S2. Number of peptides needed to capture 1%, 5, 10 and 50% of epitopes detected by mass spectrometry in B721.221 and PBMC cell lines** **using a logistic regression model.** The lower the number of peptides needed to capture the respective number of epitopes, the better the performance of the prediction model. This is also illustrated by the percentage of false identification (false positive rate, FPR) reported here. Peptides were rank ordered according to regression scores. Of note, only the maximal transcript expression was used for peptides with multiple potential origins.

| **Cell lines** | **Model** | n | FPR | n | FPR | n | FPR | n | FPR |
| --- | --- | --- | --- | --- | --- | --- | --- | --- | --- |
|  |  | 1% (n=46) | | 5% (n=231) | | 10% (n=462) | | 50% (n=2312) | |
| **B721.221**  # MAPs: 397,939  # hits:  4625  % hits/entries: 1.16 | NetMHCpan4.0 | 206  ± 25 | 77.4% | 1113  ± 43 | 79.2% | 2432  ± 109 | 81.0% | 29118  ± 530 | 92.1% |
|  | NetMHCpan4.0+ expression | 99  ± 15 | 52.6% | 473  ± 22.9 | 51.1% | 974  ± 24 | 52.5% | 8510  ± 88 | 72.8% |
|  | **NetMHCpan4.0+ expression**  **+ CAMAP** | **80**  **± 9** | **41.7%** | **440**  **± 16** | **47.5%** | **881**  **± 20** | **47.5%** | **8309**  **± 118** | **72.2%** |
|  | | 1% (n=16) | | 5% (n=81) | | 10% (n=162) | | 50% (n=808) | |
| **PBMCs**  # MAPs: 411,468  # hits: 1615  % hits/entries: 0.39 | NetMHCpan4.0 | 104  ± 24 | 83.6% | 818  ± 55 | 90.1% | 1862  ± 111 | 91.3% | 22251  ± 976 | 96.4% |
|  | NetMHCpan4.0+ expression | 145  ± 14 | 88.8% | 438  ± 34 | 81.4% | 923  ± 66 | 82.4% | 12731  ± 530 | 93.6% |
|  | **NetMHCpan4.0+ expression**  **+ CAMAP** | **98**  **± 13** | **83.4%** | **331**  **± 23** | **75.4%** | **731**  **± 39** | **77.8%** | **12365**  **± 549** | **93.5%** |
